# Bacteriological and Physicochemical Quality of Drinking Water in Adis Kidame Town, Northwest Ethiopia

**DOI:** 10.1155/2021/6669754

**Published:** 2021-02-10

**Authors:** Baye Sitotaw, Mulu Geremew

**Affiliations:** Department of Biology, Bahir Dar University, P.O. Box 79, Bahir Dar, Ethiopia

## Abstract

Drinking water pollution and the resulting waterborne diseases have been among the major public health burden in low-income countries such as Ethiopia. A high prevalence of waterborne diseases, up to 65%, has been reported in Adis Kidame Town (Ethiopia). Moreover, there have been poor waste disposal practices in this town. Thus, this study aimed to assess bacteriological and physicochemical drinking water quality in Adis Kidame Town to get insight into any potential health risks due to waterborne diseases. A total of 90 water samples were analyzed for enumeration of coliform bacteria and basic physicochemical parameters. In this study, total and faecal coliform counts (CFU/100 ml) ranged from 0 to 23 and 0 to 18, respectively. In all cases, 89 and 77% of the water samples in terms of total and faecal coliforms, respectively, did not comply with WHO guidelines. The highest level of coliforms was found in drinking water samples from households' containers and taps, where 100% and 90% of samples were tested positive for total and faecal coliforms, respectively. Among the physicochemical parameters recorded, only temperature and residual chlorine did not comply with WHO guidelines. Temperature values in all samples ranged from 20 to 28°C, while the values of residual chlorine were below the recommended range in the 56.7% of water samples from the reservoir and almost in all (96.7%) water samples from the taps and households' containers. High coliform count in the water system demands proper chlorination, regular inspection of the distribution line, and good hygienic practices to improve the microbiological quality of drinking water in Adis Kidame Town.

## 1. Introduction

Access to clean water and sanitation services for all, which is stated in Goal 6 of the Sustainable Development Goals (SDGs), has been endorsed by all countries in the world to achieve SDGs. Despite some improvements, public health burden related to drinking water contamination and low water supply has still been affecting communities in low-income countries such as Ethiopia. Drinking water can be polluted at any point in the chain from the source to household container and polluted water, in turn, can carry several waterborne pathogens [1]. Consequently, water-related diseases have been affecting 80% of the world population and about 2 billion people use faeces-contaminated water that, in turn, caused an estimated 485000 diarrhoeal deaths each year [2, 3]. These problems have been the worst among sub-Saharan countries mainly due to several sociodemographic and economic factors [4, 5].

In Ethiopia, basic water and sanitation services are very low [6] resulting in a high prevalence of water-related diseases. Safe drinking water coverage is about 66%; only 6.3% of households have access to improved sanitation, and over 83 million people live in unhealthy environments. As the result, water-related diseases accounted for 60–80% of all illnesses and diseases in Ethiopia [7, 8].

Several studies conducted in Ethiopia revealed an extremely high rate of drinking water contamination [9–13]. However, no study was conducted in Adis Kidame Town (northwest Ethiopia). Like in most other towns in Ethiopia, the environmental sanitation, hygiene situation at the household level, and improper waste disposal systems in this town were observed to be very poor. Owing to this, most of the patients visiting the health facilities were found to be affected by waterborne diseases (official reports of the health sector of the town). This study thus aimed to assess the bacteriological and physicochemical quality of drinking water in this town to get insights into the extent of health risks due to drinking water pollution.

## 2. Materials and Methods

### 2.1. Study Area Description

This study was conducted in Adis Kidame Town, Faggeta Lekoma District, northwest Ethiopia. This town is located some 459 km northwest of Addis Ababa (the capital city of Ethiopia), at the latitude of 11°04N and longitude of 36°53E, and at elevation of about 2400 meters above sea level. The area is in a midland climatic zone where the annual mean temperature and rainfall were 11–25°C and 2379 mm, respectively. According to a 2018/2019 report of Addis Kidame Town administration office, the town has an estimated total population of 17,189 of which 42.58% were males and 57.41% were females. In the town, there were 2718 households of which 2540 use tap water and the rest 178 use well water. Based on the town's health center report for 2019, the prevalence of waterborne diseases in the town was 65.3%, and the most common waterborne diseases were typhoid fever, diarrhea, amoebiasis, and functional diarrhea.

The drinking water system in Adis Kidam Town is established based on basic requirements. The source of drinking water (a developed spring) is relatively in a good sanitary status, where the surrounding area was covered with vegetation and with little human activity in the area. The reservoir is also properly built and situated in the uphill area of the town with good vegetation cover. Chlorination of the water system in this town is done at the reservoir using calcium hypochlorite powder (at the concentration of 2.5 mg/L of water), which contains 65–70% available chlorine. However, like in many areas in Ethiopia, chlorination was performed inappropriately. Based on the information obtained from the responsible person (personal communication) as well as authors' observation, less contact time, irregular chlorination, frequent breakage in the distribution line, and not checking free chlorine before distributing the water were major limitations.

### 2.2. Study Design and Parameters

A cross-sectional study was conducted in Adis Kidame Town from February to June 2020 to assess the quality of drinking water. Thirty households who used piped water were selected by systematic random sampling technique. Water samples from the reservoir, taps, and households' containers were collected for the analyses of coliforms and major physiochemical parameters. Besides, visual assessment and structured questionnaires were conducted to assess the sanitary status and risk factors for water quality.

### 2.3. Sample Size, Sampling, and Sample Handling

A total of 90 water samples, 30 samples from each of the reservoir, taps, and households' container, were collected. One litre of the water sample was collected aseptically using sterilized bottles for bacteriological examination and acid-washed glass bottles for nitrite and residual chlorine analyses. The samples were taken in the morning between 7.00 and 8.00 am, kept in an icebox and transported to the water quality laboratory (Awi Zone Water Supply and Development Sector, Ethiopia) within 2 hours, and analyzed immediately after arrived at the laboratory.

### 2.4. Enumeration of Coliforms

Membrane filtration technique was used for coliform counts following the procedures described in APHA [14]. Aseptically, the absorbent pads were placed into the petri plates and saturated with the Lauryl Sulphate Broth (HiMedia). One hundred millilitre of the water sample was filtered through a 0.45 *µ*m membrane filter (HACH company), and the filter papers were put onto the absorbent pad. The plates were then incubated at 37°C and 44°C for 24 hr for total coliform and faecal coliform counts, respectively. Colonies with characteristic colors for both total and faecal coliforms were counted using the colony counter.

### 2.5. Determination of Physicochemical Parameters

The following basic physicochemical parameters were determined in all samples following the procedures described in APHA [14]. Temperature (Bio Abron student mercury thermometer, pH (Wagtech pH meter, model CP 1000, Singapore), turbidity (Wagtech turbidity meter (model Wag-WT 302, Singapore), and TDS and conductivity (TDS/conductivity meter, Wagtech 534000, Singapore) were measured *in situ.* Residual chlorine and nitrite were determined by photometric methods using Palintest Photometer 7500 (Wagtech, Thatcham, Berkshire, UK).

### 2.6. Sanitary Assessment of the Drinking Water System

A structured questionnaire was used to obtain information about sociodemographic characteristics and sanitary status of the 30 households. The questionnaires were first developed in English and translated into Amharic (local language), and then the responses were translated back into English. In addition, visual sanitary inspection was conducted on the water source, the reservoir, and the distribution lines based on WHO checklists (Supplementary data).

### 2.7. Data Analysis

Data were analyzed using SPSS statistical software (version 23). Significant differences in the mean values of measured parameters among the different drinking water samples were tested using the *t*-test. The values of mean bacterial counts and physicochemical parameters were compared with WHO guidelines, which is more or less similar to the Ethiopian standards for drinking water quality. In addition, water samples were categorized into the different risk levels according to the risk classification for thermotolerant coliforms suggested by Tadesse [15]. In all cases, statistical significance was considered at a 95% confidence interval and a *p* value of ≤0.05.

### 2.8. Limitations of the Study

The presence of *E. coli* was not tested while this is a highly recommended microbial indicator for recent faecal pollution. We also understand that self-administered questionnaires usually bias the results and the interpretations that follow.

## 3. Results

### 3.1. Bacteriological Quality of Drinking Water in Adis Kidame Town

In this study, 89% (80/90) and 77% (69/90) of the water samples were tested positive for total coliform (TC) and faecal coliform (FC), which means that they did not comply with the WHO standard or not acceptable for drinking (Table 1). Variation in the mean counts of coliforms among the different water samples was significant (*p* < 0.05), except for FC counts between taps and households' containers. The highest (9.50 CFU/100 ml) and the lowest (1.9 CFU/ml) mean TC counts were recorded in the water samples taken from households' containers and water samples taken from the reservoir, respectively (Table 1). Comparing each sampling point, 100% (30/30), 97% (29/30), and 70% (21/30) of the households' containers, taps, and reservoir, respectively, were tested positive for TC. Likewise, 90% (27/30) of water samples from taps or households' containers, and 50% (15/30) of the water samples from the reservoir were tested positive for FC.

According to risk classification for faecal coliforms [15], mean value faecal coliforms (FCs) in the taps and households' containers were in the *low-risk* categories (Table 2). However, based on FC count in all samples, 10 and 80% of water samples from taps and households' containers, respectively, were at *high-risk* and *low-risk* categories. Only 10% (3/30) of water samples from households' containers and the taps were at *no-risk* category. Regarding the reservoir, half of the samples were at *no-risk* category while the rest half were at *low-risk* category.

### 3.2. Physicochemical Drinking Water Quality in Adis Kidame Town

Value for all physicochemical parameters, except for temperature and residual chlorine, of drinking water in Adis Kidame Town was found to be within the permissible limits set by the WHO (Table 2). The recorded temperature values for all samples were above the permissible limit while the recorded residual chlorine values were below recommended ranges in all water samples from taps and households' containers. Even residual chlorine values were below the recommended ranges in the 57% (17/30) of water samples taken from the reservoir. The recorded mean temperature and residual chlorine values were not within the recommended limit set in WHO guidelines (Table 2).

### 3.3. Sanitary Survey at the Source, Reservoir, Distribution Line, and Household Level

Generally, the terrain (the slope of the land) of the study area is almost flat. In Adis Kidame Town, some poorly constructed or damaged latrines, construction activities, farming activities including pesticides and fertilizers use, sedimentation due to flooding, wastes that were thrown everywhere, and oily wastes from cars and garages were commonly observed.

The source of drinking water for this town is a developed spring which is far from the town and relatively in a good sanitary status. The area around and uphill of the source was covered with vegetation and there was little human activity in the vicinity. The reservoir is located uphill to the town, and the area around the reservoir is almost covered with vegetation. Moreover, the reservoir is built properly and likely cannot be damaged by rainfall easily; no animal access to the water reservoir was observed; no household wastes were dumped near or uphill of the reservoir, and no evidence of discharge of sewage to the reservoir areas was found. However, the presence of human feces/open defecation near the reservoir was observed. Breakage and leakage of water in the water distribution system from the reservoir to the taps can frequently be observed.

A total of 18 questions were presented to the selected households to obtain information on the sociodemographic characteristics and sanitary conditions at the household level (Table 3). Of the respondents, a significant proportion of them did not wash their hands before drawing water from storage (90%), covered water container inadequately (83.3%), washed their water container with water only (63.3%), retained wastes at home for more than 2 weeks (66.7%), and used buckets as water storages (53.3%).

## 4. Discussion

Waterborne diseases have been persistent public health challenges in Ethiopia. According to the clinical information and sanitary status in Adis Kidame Town, a high risk of drinking water pollution was apparent in this town. This study assessed the quality of drinking water from the reservoir to customers' containers and the associated risk factors in Adis Kidame Town.

The detection of faecal coliform is a strong evidence for faecal contamination, while a high level of total coliform at least implies inadequate chlorination that in turn could lead to bacterial contamination of the drinking water. Almost all water samples (89%) for total coliforms and 77% of the samples for faecal coliforms, respectively, failed to comply with the WHO and Ethiopian guidelines for drinking water quality [1, 16]. Similarly, other studies in Ethiopia reported a high rate of total coliform prevalence of 77–100% [9–11] and 80 to 100% prevalence of faecal coliform [10–13] in the drinking water system. Drinking water pollution at the source, distribution line, or the household level has been a big public health concern in developing nations such as Ethiopia [12–16]. In this study, water samples from almost all the households' containers were found to be contaminated with coliforms, which strongly suggested poor handling practices at the household level. Likewise, most samples from taps were contaminated with coliforms, which is likely due to the damage in the waterline to the taps and the possible entry of pollutants.

Cleaner environments and hygienic households are compulsory to ensure potable water. Relatively the source of drinking water and the reservoir in Adis Kidame Town were located at a good sanitary status. In contrast to this, the sanitation status in Adis Kidame Town in particular and Ethiopia as a whole is very low which was 52.1% during 2014 and far lower than the national plan set to reach 60% by 2015 [7]. Although the sanitary statuses around the sources and reservoir were relatively good, open defecation, poor waste disposal system, and agricultural activities in the town were common. In addition, drinking water handling practices in the community of Adis Kidame Town were poor where most of the respondents had poor handwashing habit before drawing water from storage, not properly washed the water container as well as used unsafe water container (bucket) and poor waste disposal habit (Table 3).

Regarding the recorded physicochemical water quality parameters, the Adis Kidame Town drinking water system can be considered safe. However, the temperature recorded was higher than the recommended limit value, and this can be conducive for the proliferation of aerobic mesophilic bacteria and, in turn, may contribute to the high bacterial counts recorded. Drinking water temperature above the aesthetic objective value (15°C) increases the taste intensity and odor of the water, which reduce acceptability. In fact, in most similar studies elsewhere in Ethiopia, the temperature record has been higher than the standard limit, even in water systems where the pollution level was very low [17]. This suggests that the temperature limit value in the tropics may need a revision. The residual free chlorine concentration after 30 min of addition is expected to be ≥0.5 mg/L at pH <8.0 [15]. The residual chlorine level in the samples was found to be far less than the recommended range implying a high risk of contamination. Where adequate water treatment is not done, the impact on public health can be devastating due to pathogenic bacteria contamination.

## 5. Conclusions

Bacterial drinking water quality in Adis Kidame Town fails to comply with WHO guidelines. The most important risk factors for water quality deterioration in Adis Kidame Town include frequent breakage in the distribution line, poor waste disposal system in the town, agricultural activities that use pesticide and herbicide, and unhygienic water handling practices at the household level. In order to reduce waterborne health burden, the town administration in cooperation with responsible offices and the community should take immediate actions including proper chlorination, regular inspection and maintenance of the waterline, environmental sanitation, limiting excessive use of agricultural inputs, and most importantly educating the community on how to properly handle drinking water at the household level.

## Figures and Tables

**Table 1:** Coliform counts (CFU/ml) in the drinking water samples from the reservoir, taps, and households' containers in Addis Kidame Town, 2020.

Indicators	Codes	Description	Mean ± SD	Range	Risk levels
TC	R	Reservoir/disinfection point	1.9 ± 1.6	0–6	—
	T	Taps at each household	6.1 ± 4.5	0–18	—
	H	Households' containers	9.5 ± 5.2	3–23	—
FC	R	Reservoir/disinfection point	0.7 ± 0.9	0–4	NR
	T	Taps at each household	4.4 ± 4.2	0–14	LR
	H	Households' containers	4.6 ± 4.3	0–18	LR

*Note*. TC = total coliform count, FC = faecal coliform count, LR = *low-risk*, and NR = *no-risk*.

**Table 2:** Mean (*n* = 30) physicochemical values of drinking water samples from the reservoir, taps, and households' containers in Addis Kidame Town, 2020.

Sample sources	Parameters					
	pH	Temperature (°C)	Turbidity (NTU)	Conductivity (*μ*s/cm)	NO_2_ (mg/l)	Res. Cl_2_ (mg/l)
R	6.7 ± 0.04	22.5 ± 1.1	0.7 ± 0.3	167.5 ± 7.2	0.03 ± 0.05	0.16 ± 0.1
T	7.7 ± 0.1	22.6 ± 1.2	0.9 ± 0.7	156.1 ± 5.5	0.02 ± 0.02	0.06 ± 0.05
H	7.7 ± 0.2	22.9 ± 1.6	0.7 ± 0.4	152.4 ± 12.6	0.017 ± 0.02	0.08 ± 0.1
WHO	6.5–8.5	<15	<5	1000	3	0.2–0.5

*Note*. R = reservoir, T = taps, and H = households' containers.

**Table 3:** Sociodemographic characteristics of the participant and some other risk factors for drinking water pollution in Addis Kidame Town, 2020.

Risk factors	Categories	Frequency	Percentage
Sociodemographic characteristics of the households			
Sex of the head	Male	12	60.0
	Female	18	40.0

Age of the respondents	18–30	14	46.7
	31–45	11	36.7
	>45	5	16.7

Educational status of the family head	Primary school	13	43.3
	Secondary school	13	43.3
	College and above	4	13.3

Occupation of the family head	Farming	6	20
	Daily laborer	6	20
	Gov. employee	14	46.7
	Merchant	4	13.3

Family income in ETB birr	500–000	2	6.7
	1000–000	10	33.3
	3001–000	10	33.3
	>5000	8	26.7

Family size	2–4	14	46.7
	5	4	13.3
	>5	12	40

Other risk factors			
Source of drinking water	Privet pipe	29	96.7
	Neighbor pipe	1	3.3
What types of water container do you use to fetch?	Bucket	11	36.7
	Clay pot	5	16.7
	Jerry can	14	46.7

What type of storage do you use?	Bucket	16	53.3
	Clay pot	1	3.3
	Jerry can	13	43.3

How often do you properly wash water storage?	Rarely	7	23.3
	Sometimes	1	3.3
	Always	22	73.3

Type of cleaning agent you use to wash the container	Ash or *Grawa*	6	20.0
	Detergent/soap	5	16.7
	Only water	19	63.3

Approximate distance of tap water from the toilet	>8 m	2	6.7
	3–6 m	19	63.3
	6–8 m	9	30.0

Frequency of removing wastes from your compound	1-2 weeks	8	26.7
	<1 week	2	6.7
	>2 weeks	20	66.7

Have you ever observed any damage observed on the part of tape water?	No	22	73.3
	Yes	8	26.7

Have you ever observed any damage on the part of the pipe?	No	27	90.0
	Yes	3	10.0

Do you wash your hands before drawing water from storage?	Yes	3	10.0
	No	27	90.0

Is the water container inadequately covered?	Yes	25	83.33
	No	5	16.7

What is the approximate distance of tap water from your home?	>3 m	6	20
	3–6 m	8	26.7
	>6 m	16	53.3

## Data Availability

The data used to support the findings of this study are included in the paper and Supplementary data.

## Supplementary Materials

Supplementary Materials Supplementary data 1: observational checklist for sanitary conditions in Adis Kidame Town, around the water source, reservoir, and the distribution line.
