# Supplementary material for: Bacteriological and Physicochemical Quality of Drinking Water in Adis Kidame Town, Northwest Ethiopia
Source: Int J Microbiol. 2021 Feb 10;2021:6669754. doi: 10.1155/2021/6669754 (PMC7889384; doi:10.1155/2021/6669754)
Supplement: Supplementary Materials — Supplementary data 1: observational checklist for sanitary conditions in Adis Kidame Town, around the water source, reservoir, and the distribution line. [file 6669754.f1.doc]

Supplementary data 1: observational checklist for sanitary conditions in Adis Kidame town, around the water source, reservoir and the distribution line.

| **No.** | **The checklist** | **Observation** |
| --- | --- | --- |
| **A. Sanitary situation of the town in general** | | |
| 1 | The terrain (the slope of the land) of the town | Almost flat |
| 2 | **Presence of poorly constructed/damaged latrines** | **yes** |
| 3 | **Construction activities in the town** | **yes** |
| 4 | **Farming activities including pesticides and fertilizers use** | **yes** |
| 5 | **Sedimentation due to flooding** | **yes** |
| 6 | **Wastes disposal situation** | **observed thrown everywhere** |
| 7 | **Presence of oily wastes from cars and garages** | **yes** |
| 8 | Open defecation was observed | yes |
| **B. The sanitary situation of the water source** | | |
| 9 | Location of the source of drinking water | Fairly far from the town |
| 10 | Vegetation cover of the area around and uphill of the sources | yes |
| 11 | Human activity in the vicinity of the source | minimal |
| **C. The sanitary situation of the resrvior** | | |
| 12 | Location of the reservoir | uphill to the town |
| 13 | Reservoir is built properly/cannot be damaged by rainfall easily | yes |
| 14 | The reservoir is properly fenced | yes |
| 15 | Vegetation cover around the reservoir | yes |
| 16 | Animal access to the water reservoir was observed | no |
| 17 | Household wastes were dumped near or uphill of the reservoir, | no |
| 18 | Evidence of discharge of sewage to the reservoir area | no |
| 19 | **Presence of human faeces/ near to the reservoir** | **yes** |
| 20 | **Breakage and leakage of water in the water distribution line from reservoir to the taps** | **yes** |
